# The critical roles of three sugar-related proteins (HXK, SnRK1, TOR) in regulating plant growth and stress responses

**DOI:** 10.1093/hr/uhae099

**Published:** 2024-04-04

**Authors:** Guangshuo Li, Ying Zhao

**Affiliations:** College of Enology and Horticulture, Ningxia University, Yinchuan 750021, China; Section for Ecology and Evolution, Department of Biology, University of Copenhagen, 2100 Copenhagen East, Denmark; College of Enology and Horticulture, Ningxia University, Yinchuan 750021, China

## Abstract

Sugar signaling is one of the most critical regulatory signals in plants, and its metabolic network contains multiple regulatory factors. Sugar signal molecules regulate cellular activities and organism development by combining with other intrinsic regulatory factors and environmental inputs. HXK, SnRK1, and TOR are three fundamental proteins that have a pivotal role in the metabolism of sugars in plants. HXK, being the initial glucose sensor discovered in plants, is renowned for its multifaceted characteristics. Recent investigations have unveiled that HXK additionally assumes a significant role in plant hormonal signaling and abiotic stress. SnRK1 serves as a vital regulator of growth under energy-depleted circumstances, whereas TOR, a large protein, acts as a central integrator of signaling pathways that govern cell metabolism, organ development, and transcriptome reprogramming in response to diverse stimuli. Together, these two proteins work to sense upstream signals and modulate downstream signals to regulate cell growth and proliferation. In recent years, there has been an increasing amount of research on these three proteins, particularly on TOR and SnRK1. Furthermore, studies have found that these three proteins not only regulate sugar signaling but also exhibit certain signal crosstalk in regulating plant growth and development. This review provides a comprehensive overview and summary of the basic functions and regulatory networks of these three proteins. It aims to serve as a reference for further exploration of the interactions between these three proteins and their involvement in co-regulatory networks.

## Introduction

In plants, nutrient signaling mechanisms are complex and form part of the regulatory networks, providing distinct physiological, metabolic, and functional regulation in different cells and tissues. Nutrient signals regulate cellular activities and organism development by integrating with other intrinsic regulatory factors and environmental inputs [1, 2]. The sugar metabolic network is one of the most critical regulatory networks in nutrient signaling mechanisms and plays a vital role in important biological processes within plants [2]. As an essential energy source and signaling molecules, sugars play a crucial role in the sugar metabolic network [3]. Hexokinase (HXK), SNF1-related protein kinases 1 (SnRK1), and target of rapamycin (TOR) serve as crucial sugar response signals that play a regulatory role in the growth of plants and respond to external nutrients and light [2, 4–9].

**Figure 1:**
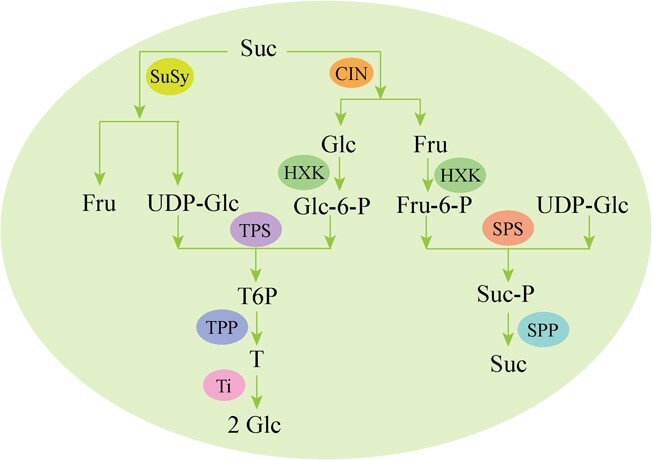
Sugar metabolism in plants. Sugar metabolism in plants. Suc, sucrose; SuSy, sucrose synthases; Fru, fructose; Glc, glucose; HXK, hexokinase; CIN, cytoplasmic invertases; T6P, trehalose-6-phosphate; TPS, trehalose-6-phosphate synthase; TPP, T6P phosphatase; Ti, trehalase; Suc-P, sucrose phosphate; SPS, sucrose P synthase; SPP, sucrose P phosphatase.

Sucrose, the main carbohydrate produced through photosynthesis in higher plants, is transported through the phloem to different tissues. In these tissues, it is hydrolyzed by cytoplasmic invertases (CIN) into glucose and fructose (Fig. 1) [5, 10–12]. Meanwhile, sucrose can be converted into UDP-glucose and fructose by the action of sucrose synthases (SuSys). Glucose and fructose are converted into glucose-6-phosphate (Glc-6-P) and fructose-6-phosphate (Fru-6-P) by HXKs [1, 11]. UDP-glucose and Glc-6-P undergo enzymatic conversion mediated by trehalose-6-phosphate synthase (TPS), resulting in the formation of trehalose-6-phosphate (T6P). Subsequently, T6P is further transformed into trehalose via the action of T6P phosphatase (TPP). Finally, the trehalose can be hydrolyzed by trehalase into two molecules of glucose. Fru-6-P and UDP-glucose are converted into sucrose phosphate (Suc-P) by Suc-P synthase (SPS), and then Suc-P is converted into sucrose by the action of Suc-P phosphatase (SPP) (Fig. 1) [11].

The above sugar molecules are able to regulate the receptors. For example, Glc-6-P and T6P inhibit SnRK1 [1, 13, 14]. Glucose activates TOR and HXK1 proteins to regulate growth and development [1, 3, 15, 16]. Apart from their participation in the modulation of sugar signaling, HXK, SnRK1, and TOR also serve as pivotal regulators of stress responses that mediate plant growth and development. HXKs are recognized for their capacity to elicit responses in the face of salt and drought stress conditions [3]. SnRK1 participates in the signaling cascade of abscisic acid (ABA), contributing to its regulatory role in plant physiological processes [17, 18]. TOR is a well-known protein that coordinates plant growth [19, 20]. The regulatory network of sugar signaling molecules is important and complex. In recent years, there has been increasing research on these three proteins, revealing that they not only regulate sugar signaling but also exhibit certain signal crosstalk in the regulation of plant growth and development processes. Currently, most reviews focus on the two sugar proteins TOR and SnRK1, and less information about HXK has been summarized. The potential signal crosstalk among these three proteins has also been less explored. This article provides an overview of the significant significance and relationships of these three proteins in regulating plant sugar metabolism and stress responses, aiming to provide valuable insights into understanding plant growth and development processes.

## Hexokinases

Hexose phosphorylation is a crucial and irreversible reaction in the early stages of sugar metabolism in plants [21]. Enzymes that facilitate this reaction are classified into three categories based on their affinity for substrates: fructokinases (FXKs), glucokinases (GLKs), and HXKs [21]. Although both GLKs and FRKs participate in the metabolism of glucose and fructose, respectively, they exhibit specificity towards their respective substrates. HXKs can phosphorylate a range of hexose sugars, including glucose, fructose, mannose and galactose [21, 22]. The HXK protein family is widely present in plants and plays a significant role [23–25]. Grape, for example, is known for high sugar content, and the HXK protein family has also been identified. Researchers have identified six genes encoding VvHXK proteins in *Vitis vinifera* by using a comprehensive screening approach on the grape genome sequence [21, 26]. The six *VvHXK* genes were localized on four chromosomes [21]. All of the members of the *VvHXK* gene family comprise 9 coding exons, which are interrupted by 8 introns, with the exception of *VvHXK5*, which comprises 10 exons and 9 introns, and *VvHXK2*, which consists of 4 exons and 3 introns [21]. An investigation into the *cis*-acting elements present in the promoter region of six *VvHXK* genes has revealed a diverse range of *cis*-acting elements that can be regulated by various factors, including light, sugar, plant hormones, and abiotic stress [21]. This implies that the relationship between the HXK family and plant growth and development requires further exploration.

### Hexokinase signaling in plant stress responses

The function of HXK is vital in plant responses to different stresses, such as biotic stress resulting from pathogen infections [27]. The predominant fungal pathogen responsible for infecting peach fruit is *Monilinia fructicola*, which leads to the development of brown rot and postharvest decay [27]. Researchers discovered that infection by *M. fructicola* caused a rise in the activity of HXK and gene expression of *HXK2*, which in turn led to a faster breakdown of sucrose in peach fruit (*Prunus persica*). Furthermore, a positive relation was found between the activity of HXK and the expression of the *PpHXK2* gene, and there was a negative correlation between the activity of HXK and the sucrose content within the fruit [27]. The presence of three W-box *cis*-acting elements within the *PpHXK2* gene promoter suggests that PpHXK2 potentially acts as a sugar sensor, playing a role in the process of sugar signaling and metabolism in the context of immune responses to pathogen infections [27]. The expression of *Nicotiana benthamiana*  *HXK1* displayed an increase in expression levels in tobacco leaves when infected with *Pseudomonas syringae*. Moreover, it was observed that a high level of HXK significantly enhanced the plant’s ability to resist the infection [28]. *BnHXK1*, *BnHXK3*, and *BnHXK9* have been identified in *Brassica napus* as participating in the defense response against *Sclerotinia sclerotiorum* pathogen [29]. Several studies have demonstrated that the reduced activity of HXK can lead to decreased resistance against *M. fructicola* infection by influencing sugar metabolism and the phenylpropanoid biosynthetic pathway [30].

HXK plays a crucial role in regulating plant drought resistance, and it has been found that GNC (GATA, NITRATE-INDUCIBLE, CARBON METABOLISM INVOLVED), which belongs to the GATA family of transcription factors, is involved in this process [31]. Studies have revealed that *PdGNC* activates *PdHXK1*, resulting in a substantial increase in HXK activity. This activation enhances the plant’s ability to cope with water shortage by improving sugar metabolism and other related physiological processes [31]. Furthermore, HXK facilitates the generation of nitric oxide (NO) and hydrogen peroxide (H_2_O_2_) within guard cells, leading to a reduction in stomatal opening and ultimately enhancing the plant’s resistance to drought [31]. A similar result was found in strawberry (*Fragaria pentaphylla*). Inhibition of FpHXK1 kinase activity weakened the strawberry plant’s responses to drought stress [25]. Meanwhile, about HXK2, researchers found that *Arabidopsis* plants transgenic for *Glycine max HXK2* (*GmHXK2*) showed improved tolerance to salt stress [24]. Conversely, *GmHXK2* ­silenced plants showed decreased expression of genes associated with salt tolerance, resulting in increased sensitivity to salt stress [24]. At same time, the study showed a positive correlation between the *GmHXK15* gene and HXK activity. Furthermore, when *GmHXK15* was overexpressed in soybean hairy roots, it led to increased tolerance to alkali stress [32].

By ectopically expressing the *Prunus HXK3* gene, an improvement in the tolerance of *Arabidopsis* to both salt and drought stress was observed under the conditions of a growth chamber [33]. In addition, metabolomics analysis of these transgenic *Arabidopsis* plants indicated alterations in the levels of several metabolites, such as G6P (phosphorylated sugars), starch, and certain metabolites linked to the TCA (tricarboxylic acid) cycle [33]. By identifying specific metabolic changes associated with the transgenic *Arabidopsis* plants, this establishes a robust basis for subsequent investigations focused on understanding how the gene functions in imparting stress tolerance. This information could be useful in further studies aimed at understanding the mechanisms by which the *Prunus HXK3* gene confers salt and drought tolerance, as well as in identifying other genes and pathways that may be involved in these processes [33]. *HXK*s responded to cold stress in *Jatropha curcas* [34]. Specifically, *JcHXK1* and *JcHXK2* were found to be upregulated in the leaves after being exposed to cold at a temperature of 12°C for 12 and 24 h [34]. In the root, *JcHXK1*, *JcHXK2*, and *JcHKL1* (*HXK-like*) exhibited comparable downregulation patterns following 12 and 24 h of exposure to 12°C cold stress [34].

## SNF1-related protein kinase 1

**Figure 2:**
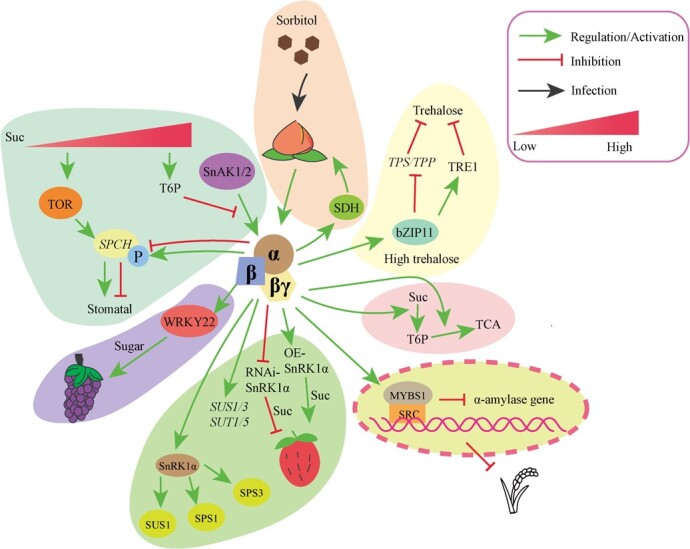
SnRK1 signaling in sugar metabolism. SnRK1 signaling regulates plant sugar metabolism. SnAK1/2, SnRK1 activate kinases 1/2; SPCH, *SPEECHLESS*; P, phosphorylation; SDH, sorbitol dehydrogenase; TPS, trehalase phosphate synthases; TPP, trehalose phosphate phosphatase; TRE1, trehalose metabolism related gene expression; bZIP11, basic leucine zipper 11; TCA, tricarboxylic acid cycle; MYBS1, MYB transcription factor; SRC, sugar response complex; OE, overexpression; RNAi, RNA interference; SUS, sucrose, synthase; SUT, sucrose transporter; SPS, sucrose phosphate synthase; WRKY, transcription factor.

SNF1-related protein kinase 1 (SnRK1) serves as a widely conserved key regulator of plant growth, playing a critical role in the maintenance of energy homeostasis during periods of limited energy availability. In mammals and yeast, its functional counterparts are represented by AMPK and Snf1 kinases, respectively [35]. SnRK1 is a prototypical heterotrimeric protein complex found in plants, consisting of an α kinase subunit and β and βγ regulatory subunits [1, 35, 36] (Fig. 2). *Arabidopsis* encodes two types of α catalytic subunit, classified as SnRK1.1 (KIN10) and SnRK1.2 (KIN11) [37]. In the yeast *snf1* mutant, the SnRK1α subunit from different plants (*Arabidopsis*, tobacco, potato, rice and wheat) could functionally complement, which indicated a conserved functional role across eukaryotes [35, 38–42].

### SnRK1 signaling in plant sugar metabolism

The application of sorbitol in peach fruits increased the activity of certain enzymes, such as SnRK1, sucrose synthase (SS), and sorbitol dehydrogenase (SDH), leading to an increase in sucrose content compared with the control group [43] (Fig. 2). Furthermore, the interaction between SnRK1α and SDH in peach indicated the activation of SDH by SnRK1, facilitating sorbitol metabolism and regulating the activity of SS. This, in turn, facilitated the accumulation of sucrose in peach fruit [43] (Fig. 2). In the presence of elevated trehalose levels, SnRK1 interacted with bZIP11 to inhibit the activity of genes related to trehalose synthesis (*TPS* and *TPP*) and activate gene expression related to trehalose metabolism. Consequently, there was a reduction in the trehalose content in peach [44]. In grape, SnRK1.1/1.2 interacted with the WRKY22 transcription factor to regulate sugar accumulation [45]. In strawberry fruit, overexpression of SnRK1α resulted in an elevation of sucrose content, while suppression of SnRK1α expression via RNA interference resulted in a decrease in sucrose content [46]. Regarding the genes associated with sugar metabolism, SnRK1α increased the expression of *sucrose synthase 1* (*SUS1*) and *SUS3* [46]. Meanwhile, the expression levels of sucrose transporter genes, namely *SUT1* and *SUT5*, was enhanced by SnRK1α. Moreover, SnRK1α interacted with proteins SUS1, SPS1, and SPS3 in strawberry in the yeast two-hybrid (Y2H) assay [46].

Through the analysis of metabolomics and transcriptomics in *Arabidopsis SnRK1* gaining and losing function mutants, researchers have discovered that SnRK1 contributes to modifying the association between T6P and sucrose. This modification impacts the pathway responsible for converting sucrose into T6P accumulation, while also regulating the carbon flow towards the downstream part of T6P signaling in the TCA cycle [47]. Scientists further explored the T6P–SnRK1 pathway and identified a new candidate gene, TPP-7A, which is specifically expressed in developing grains and significantly influences grain plumpness and size. This gene primarily regulates the breakdown, flux, and utilization of sucrose in the endosperm of grains through the T6P–SnRK1 pathway and the sugar–ABA interaction feedback mechanism [48].

SPCH (SPEECHLESS) acts as a central regulator governing the development of stomata [49, 50]. Researchers have discovered that both TOR and SnRK1 play important roles in regulating the transcriptional activity and stability of the SPCH protein, thus, affecting the development of stomata in response to exogenous sucrose supply [49]. Under conditions of low sucrose level, sucrose triggers the activation of the TOR pathway, leading to the promotion of stomatal development through the induction of SPCH expression [49]. Additionally, SnRK1 participates in this process by phosphorylating and stabilizing the SPCH protein. This phosphorylation event enhances the stability of the SPCH protein, leading to further promotion of stomatal development [49]. When exposed to elevated sucrose levels, the increased concentration of T6P inhibits KIN10 (SnRK1.1) activity by diminishing the interaction between SnAKs and KIN10. As a result, this triggers the degradation of SPCH and hinders stomatal development [49] (Fig. 2). Several studies in *Arabidopsis* have indicated that, besides KIN10 activity, other mechanisms within the SnRK1 pathway also take part in the response of elongating hypocotyls to sucrose under photoperiod/daily light conditions. For example, the low-energy response can be mediated by bZIP63. In *Arabidopsis*, KIN10 activated bZIP63 to regulate the circadian clock in the low-energy conditions [51]. Meanwhile, the enhanced expression of KIN10 caused a postponement in the maximum expression of *GIGANTEA* (*GI*), an evening element of the circadian clock, under diurnal conditions. Furthermore, it extended the duration of the clock cycle, particularly in the condition of light [52]. In the case of rice germinating embryos and cell cultures, it has been observed by researchers that sugar functions as a suppressor of α-amylase expression [42]. This repression occurs through the involvement of a sugar response complex (SRC) located in the promoters of α-amylase genes, as well as its interacting MYBS1 [42]. The sugar signaling cascade involves SnRK1α as a critical intermediary, which operates upstream of the interaction between MYBS1 and the SRC promoter for αAmy3 (α-amylase gene) (Fig. 2).

**Figure 3:**
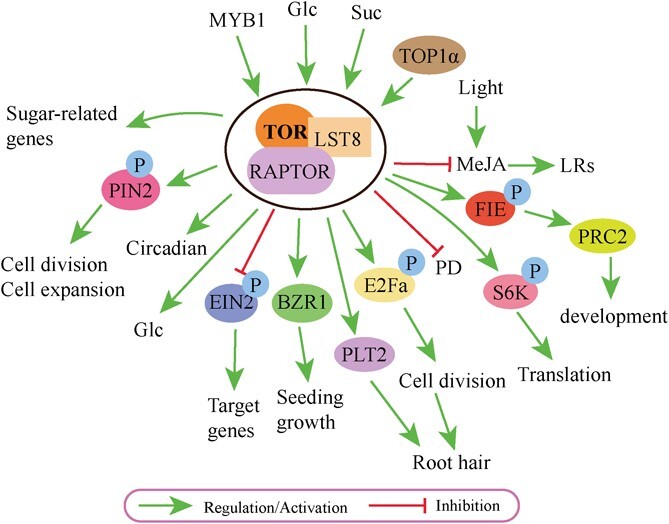
TOR signaling in sugar metabolism. Glc–TOR signaling regulates plant growth. MYB1, v-myb avian myeloblastosis viral oncogene homolog transcription factors; Glc, glucose; Suc, sucrose; MeJA, methyl jasmonate; LRs, lateral roots; S6K, 40S ribosomal S6 protein kinase; P, phosphorylation; PD, plasmodesmata; E2Fa, transcription factor; BZR1, brassinosteroid (BR) signaling transcription factor; EIN2, ethylene-insensitive protein 2; PIN2, PIN-FORMED 2; PLT, PLETHORA; PRC2, Polycomb Repressive Complex 2; TOP1α, TOPOISOMERASE1α.

## Target of rapamycin

TOR is a large protein (~280 kDa) that functions as an atypical Ser/Thr protein kinase and is highly conserved across organisms. It acts as a crucial signaling integrator, governing various cellular processes, such as organ growth, cell metabolism, and transcriptome reprogramming in response to nutrient and hormonal signals, and stress conditions [1, 53–58]. In most plants, there is only one TOR protein [35]. The TOR complex 1 (TORC1) is formed by TOR, LST8 (LETHAL-WITH-SEC13-PROTEIN 8) and RAPTOR (REGULATORY-ASSOCIATED-PROTEIN-OF-MTOR) to regulate plant growth, dynamically [1, 54] (Fig. 3). The TOR protein is formed by five domains, comprising HEAT (Huntington, EF3A, ATM, TOR) repeats, FAT (Focal adhesion target), FRB (FKBR12 and rapamycin binding), kinase, and FATC (C-terminal of FAT) [54, 59–61]. In typical scenarios, the FKBP12 protein combines with TOR and rapamycin to create an inhibitory complex, which has the ability to control the functioning of TOR. However, when the FKBP12 protein mutates, it is unable to form a stable inhibitory complex with TOR and rapamycin. This mutation hinders the normal inhibitory mechanism of the TOR pathway, resulting in the plant’s insensitivity to rapamycin [62, 63]. Based on the reasons mentioned above, scientists have developed a new generation of inhibitors of TOR, including KU-63794, Torin1/2, PP242, WYE-132, and AZD8055. By competing with ATP, these TOR inhibitors have the ability to bind with the kinase domain of TOR, leading to the inhibition of its activity [15, 20, 54, 64].

### TOR signaling in plant sugar metabolism

TOR can sense all kinds of upstream signals, like sugar, light, and hormones. It directly or indirectly regulates downstream signals to modulate cell growth and cell proliferation in plants [1, 19, 65] (Fig. 3). The Glc–TOR signaling pathway holds a pivotal position in plant growth, with glucose being a potent activator of TOR protein that governs the development of root hairs in plants [66]. Scientists have uncovered that glucose, originating from shoot photosynthesis, propels the relay of TOR signaling by means of glycolysis and mitochondrial bioenergetics, thereby regulating the activation of the root meristem [15]. In *Arabidopsis*, glucose activates TOR protein, which then phosphorylates E2Fa to promote root hair cell proliferation [15]. Glc–TOR–E2Fa is a novel regulatory pathway of plant hair development [15] (Fig. 3).

EIN2 (ethylene insensitive protein 2) has recently been confirmed as a substrate of TOR, with the ability to shuttle between the cytoplasm and nucleus, and can be directly phosphorylated by TOR, hindering its nuclear localization. EIN2 negatively regulates the expression of multiple target genes involved in the glc–TOR signaling pathway, including DNA replication, cell wall synthesis, and various secondary metabolic processes [20]. FIE (FERTILIZATION-INDEPENDENT ENDOSPERM), a crucial constituent of Polycomb repressive complex 2 (PRC2), is a target of TOR protein [67]. Glc–TOR–FIE–PRC2 signaling modulates plant development (Fig. 3) [67]. According to a recent study, glc–TOR signaling played a critical role in adjusting the circadian period [56, 68]. When glucose was externally applied, it activated TOR and shortened the circadian period [68]. Conversely, the induction of TOR silencing by estradiol resulted in a significant lengthening of the circadian period, completely blocking the recovery of the circadian period by exogenous glucose [68].

Efficient transportation of sugar from mature ‘source’ leaves to developing ‘sink’ leaves requires a rigorous regulation of sugar transport between cells, which is facilitated by plasmodesmata [64]. Researchers revealed that glc–TOR metabolic network plays a crucial role in restricting plasmodesmata transport in leaves [64]. Plant upgrowth is strongly influenced by the energy level within the cells [69]. By creating gradients, PLT (PLETHORA) plays a crucial role in defining the characteristics of the root apical meristem (RAM). Researchers demonstrated that TOPOISOMERASE1α (TOP1α) controlled the TOR–PLT2 pathway, which is responsible for maintaining homeostasis and gravitropism in the root tip in response to sugars during development [69].

The transcription factor BZR1, participating in brassinosteroid (BR) signaling, plays a crucial role in promoting growth in response to hormonal and environmental signals [70]. By regulating TOR, glucose signals stimulate the accumulation of BZR1 and promote seedling growth [70, 71] (Fig. 3). However, the effects of sugars are abolished when TOR is inactivated, and the degradation of BZR1 due to TOR inactivation is prevented by inhibiting autophagy [70]. These findings suggest a sequential process whereby cellular starvation leads to TOR inactivation, followed by BZR1 degradation and autophagy [70, 71]. Recent research has uncovered that glucose antagonizes the methyl jasmonate response through TOR signaling and inhibits the erect root architecture, inducing wider branching angles [72]. Through large-scale *Arabidopsis* mutant screening, scientists identified an auxin efflux facilitator called PIN2 (PIN-FORMED 2), which was a critical downstream regulator of glc–TOR signaling [65]. Glucose-activated TOR phosphorylated and stabilized PIN2, which in turn affected the gradient distribution of PIN2 in the primary root of *Arabidopsis*, thereby regulating cell growth [65].

Meanwhile, recent researchers found that TOR also regulated sugar metabolism in grapes [57]. Through the use of the yeast one-hybrid (Y1H) assay and dual-luciferase reporter systems, researchers have discovered that MYB1 plays a role in regulating TOR expression by controlling the TOR promoter, and is involved in glucose accumulation in grapes (Fig. 3) [73]. These findings indicate a potential interplay between MYB1, TOR, and glucose accumulation [73]. Except for glucose, sucrose also increased the activity of TOR and promoted S6K (40S ribosomal S6 protein kinase) phosphorylation. S6K is an important direct substrate of TOR. Detection of the phosphorylation level of S6K can be used to monitor the activity of TOR protein [1] (Fig. 3). During the extensive process of evolution, plants have developed a highly sophisticated TOR signaling network in response to sugar signaling, enabling them to withstand various environmental pressures and stresses [54].

## HXK, SnRK, and TOR signaling networks in plant growth and development

### The hexokinase signaling network

The function of HXK in regulating sugar metabolism in horticultural crops is well known. Pear (*Pyrus* × *bretschneideri*) is a widely cultivated and economically significant fruit globally [5]. Transcriptome analysis was employed to construct HXK expression patterns in various tissues. It was observed that *PbHXK1* exhibited preferential expression in fruits, while its expression levels were relatively lower in petals, sepals, ovaries, and buds [5]. *PbHXK1* overexpression in tomatoes led to a significant enhancement of HXK activity and a reduction in sugar content [5]. A negative regulatory function of PbHXK1 in sugar content modulation has been proposed [5]. Similar findings have been obtained in grapes, where a higher HXK activity and protein level in the initial of phases of grape development correlated with lower hexose content. However, as the grapes matured, there was a sharp decline in the activity and protein level of HXK accompanied by an increase in hexose levels [74].

**Figure 4:**
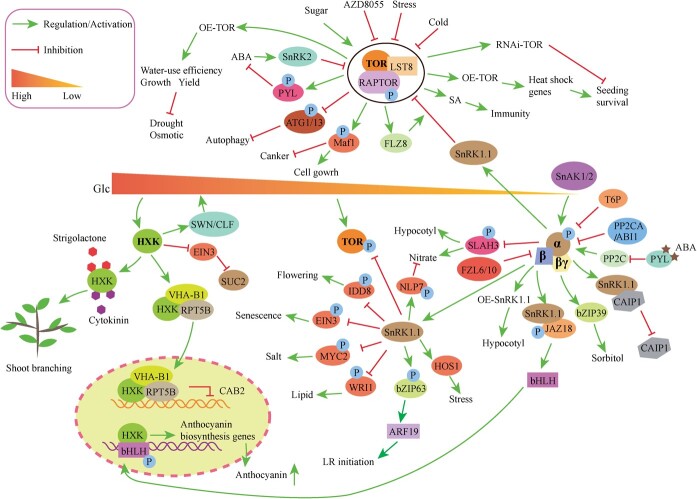
HXK, SnRK1 and TOR signaling networks in plant growth and development. HXK, SnRK1, and TOR signaling networks regulate plant growth and development. HXK, hexokinase; TOR, target of rapamycin; SnRK1, SNF1-related protein kinases 1; VHA-B1, vacuolar H^+^-ATPase B1; RPT-5B, 26S regulatory particle of proteasome subunit 5B; CAB2, chlorophyll *a/b* binding protein 2; SWN, SWINGER, two catalytic subunits of Polycomb Repressive Complex 2; CLF, CURLY LEAF; bHLH, basic helix–loop–helix; IDD8, INDETERMINATE DOMAIN 8; EIN3, ethylene-insensitive protein 3; MYC2, a bHLH transcription factor; WRI1, WRINKLED1; bZIP63, basic leucine zipper 63; ARF19, AUXN RESPONSE FACTOR; LR, lateral root; SLAH3, SLAC1 homolog 3; FLZ6/10, FCS-like zinc finger; JAZ18, a repressor in the jasmonate (JA) signaling pathway; CAIP1, C2-domain ABA insensitive protein 1; ABI1, ABA insensitive protein 1; PP2C, protein phosphatases 2C; T6P, trehalose 6-phosphate; SnAK1/2 and SnRK1 activate kinases 1/2; Maf1, a global repressor of RNA polymerase III (Pol III); ATG1/13, autophagy-related 1/13 kinase complex; PYL, a receptor of ABA; SnRK2, SNF1-related protein kinases 2; OE, overexpression; AZD8055, an inhibitor of TOR; THADA, thyroid adenoma associated; RNAi, RNA interference; SA, salicylic acid; P, phosphorylation; Glc, glucose; ABA, abscisic, acid; NLP7, NIN-LIKE PROTEIN 7; HOS1, High Expression of Osmotically Responsive Genes 1.

In addition to responding to the sugar signal, HXK can also regulate a variety of metabolic processes in plants (Fig. 4). HXK1 signaling enhances shoot branching and interacts with the cytokinin and strigolactone pathways [75]. EIN3 (ETHYL-ENE-INSENSITIVE 3) is a crucial constituent of the ethylene/glucose signaling network [76]. Researchers found that HXK1 exerts a negative influence on the stability of EIN3, and that EIN3 acts upstream of SUC2 in the modulation of root sink growth through glucose signaling [76]. Consequently, these components together formed the HXK1–EIN3–SUC2 module, which is instrumental in facilitating sucrose phloem loading in sucrose tissues, ultimately leading to an increase in sucrose content in sink roots (Fig. 4) [76]. In the condition of elevated glucose levels, AtHXK1 formed a repressor complex composed of three subunits with VHA-B1 (vacuolar H^+^-ATPase B1) and RPT5B (26S regulatory particle of proteasome subunit 5B). The trimeric complex bound to the *CAB2* (chlorophyll *a/b* binding protein 2) promoter to inhibit the transcription of *CAB2* [23, 77] (Fig. 4). The glucose-insensitive phenotype observed in *vha-B1* and *rpt5b* mutants, even in the presence of AtHXK1, was a crucial finding that suggested the essential roles of all three proteins in glucose sensing [23, 77]. In apple (*Malus domesticus*), MdHXK1 participated in regulating both anthocyanin biosynthesis and salt stress tolerance, which was mediated by glucose [16, 78]. Similarly, under high glucose concentration, MdHXK1 was shown to engage in an interaction with and subsequently phosphorylate the bHLH (basic helix–loop–helix) associated with anthocyanin biosynthesis, specifically at the Ser361 site. This phosphorylation event serves to stabilize the aforementioned transcription factor, ultimately resulting in enhanced transcriptional activity of genes participating in the biosynthesis of anthocyanin, and consequently leading to increased production of anthocyanin [16, 23] (Fig. 4). Researchers used immunoprecipitation coupled with mass spectrometry to demonstrate direct interactions between HXK1 and two catalytic subunits of the Polycomb Repressive Complex 2, SWINGER (SWN) and CURLY LEAF (CLF) (Fig. 4) [79]. These interactions were found to be instrumental in targeting shared glucose-responsive genes, thereby exerting regulatory control over glucose signaling pathways [79].

### The SnRK1 signaling network

SnRK1 is a critical player in various metabolic networks, exerting regulatory functions in multiple pathways (Fig. 4). In plants, SnRK1 is accountable for triggering comprehensive alterations in transcriptome and growth adaptation to cope with energy deprivation [1]. SnAK1/2 (SnRK1 activating kinases 1/2) play a role upstream of SnRK1 [35]. T6P, known as both a sugar molecule and a signaling molecule, functions as a key regulator in plants [54, 80]. Notably, T6P was observed to directly bind KIN10, leading to reduced interaction with upstream SnAK1/2 and consequent inhibition of phosphorylation and activation *in vitro* [1, 35, 81]. This indicates a distinctive mechanism in plants that enables SnRK1 to perceive changes in cellular metabolic status through T6P [81]. When sufficient energy is present, both PP2CA (A type 2C protein phosphatase) and ABI1 (ABA insensitive protein 1) can interact with the catalytic subunit of SnRK1, leading to dephosphorylation and inactivation of SnRK1.1 and inhibition of the SnRK1 signaling pathway [82]. However, during energy deprivation, ABA can bind to PYL (Pyrabactin Resistance1/Pyrabactin Resistance1-Like (PYL)/Regulatory Components of ABA Receptors family of ABA receptors) proteins, inhibit PP2C protein, and activate SnRK1 signaling transduction [17, 82, 83]. Signal crosstalk occurs between SnRK1.1 and ABA pathways. In apple and *Arabidopsis*, CAIP1 (C2-domain ABA Insensitive Protein 1) reduced ABA sensitivity [84]. Concurrently, SnRK1.1 interacted with CAIP1 and promoted its degradation [84]. The aforementioned research suggests that SnRK1.1 serves to inhibit CAIP1-mediated ABA sensitivity, whereas overexpression of CAIP1 partially attenuates SnRK1.1-mediated ABA sensitivity [84].

Nitrate is an important signaling molecule for plants to adapt to environmental changes. Under nitrate-depleted conditions, SnRK1.1 is activated and phosphorylates NLP7 (NIN-LIKE PROTEIN 7) at serine-125 and serine-306 sites, promoting the cytoplasmic retention and degradation of NLP7, thereby inhibiting nitrate-mediated gene expression and plant growth [85]. SnRK1.1 is also the negative regulator of SLAC1 (slow anion channel) homolog 3 (SLAH3), playing a pivotal role in the modulation of nitrate-dependent alleviation of ammonium toxicity by interacting with and phosphorylating the C terminal of SLAH3 [86]. The study also found that under dark conditions SnRK1.1 relies on the SLAH3 protein to respond to the sucrose signal and regulate hypocotyl growth [86]. Similarly, the energy signaling mediated by SnRK1 controls the elongation of hypocotyls in response to sucrose supplementation [87, 88]. These results provide a new viewpoint for studying SnRK1 in plants (Fig. 4). FCS-like zinc finger (FLZ) proteins, as adaptor proteins, facilitate the association between SnRK1 and different protein partners [89]. Specifically, FLZ6 and FLZ10 act as inhibitors of SnRK1, and their interaction takes place within cytoplasmic foci, leading to the repression of SnRK1 activity (Fig. 4) [89]. There is a relationship between SnRK1 and FLZs in regulating autophagy [90]. Recently, a study identified a clade of FLZ proteins as novel ATG8 (Autophagy related 8)-interacting partners in *Arabidopsis thaliana*. These AtFLZs inhibit SnRK1 signaling by repressing the α catalytic subunit (SnRK1.1), thereby negatively regulating autophagy. Under energy starvation, autophagy is activated to mediate the degradation of AtFLZs, thus relieving the repression of SnRK1.1 [90]. The ATG8–FLZs–SnRK1 regulatory axis forms a feedback regulation in plant autophagy [90].

SnRK1 has been identified as a crucial participant in the biosynthesis of both anthocyanins and proanthocyanidins [91–93]. Specifically, within jasmonate (JA) signaling, SnRK1.1 has been shown to interact with JAZ18 protein, ultimately phosphorylating JAZ18 and promoting its subsequent degradation via the 26S proteasome. This process then leads to the release of bHLH3, which in turn activates the expression of genes involved in the promotion of anthocyanin and proanthocyanidin biosynthesis (Fig. 4) [91]. Meanwhile, SnRK1 downregulates the biosynthesis of anthocyanin induced by high light [93]. Researchers have observed that SnRK1.1 (also known as KIN10) exerts direct phosphorylation effects on various transcription factors, thereby exerting regulatory control over a range of biological processes. Notably, SnRK1.1 has been found to reduce the protein stability of EIN3 (ethylene-insensitive protein 3) during senescence and MYC2 in response to salt. Additionally, it diminishes the transcriptional activity of IDD8 (INDETERMINATE DOMAIN 8) during flowering. Furthermore, SnRK1.1 phosphorylates WRI1 (WRINKLED1), leading to its subsequent proteasomal degradation. SnRK1.1 can also interact with HOS1 (High Expression of Osmotically Responsive Genes 1) to regulate plant responses to cold, heat, and salinity stress [94]. This multifaceted regulation by SnRK1.1 establishes the energy balance in plants [95–98]. In *Arabidopsis*, it has been observed that unforeseen darkness and short-term low light exposure disrupt energy homeostasis and diminish sugar availability in the roots, subsequently resulting in the initiation of lateral root (LR) formation [99]. ARF19 (AUXN RESPONSE FACTOR) has been identified as a pivotal regulator of LR growth [99]. SnRK1 has been found to phosphorylate bZIP6, which in turn directly binds to and activates the promoter region of ARF19 [99] (Fig. 4). SnRK1 mediated the phosphorylation of bZIP39 to regulate sorbitol metabolism in apple [100]. In rice, overexpression *OsSnRK1α* improved leaf photosynthetic activity and contributed to grain filling and panicle development [101]. *OsSnRK1α* also regulates plant immunity against pathogens [102].

### The TOR signaling network

The powerful functions of the TOR signaling pathway are gradually becoming well known. TOR functions as a pivotal regulator in controlling root branching by integrating local auxin-dependent pathways with systemic metabolic signals that modulate the translation of genes induced by auxin [19, 103]. In *A. thaliana*, the process of lateral root formation is characterized by the auxin-mediated activation of specific genes, namely ARF7, ARF9, and LBD16 [19]. The inhibition of TOR activity has been shown to diminish the translation of ARF19, ARF7, and LBD16, thereby affecting the expression of these key regulatory factors [19]. The interplay between TOR and ABA pathways is crucial for preserving a delicate balance between plant growth and stress responses [83]. RAPTOR is an essential component of the TOR complex. Under stressful conditions, ABA activates SnRK2s, which subsequently phosphorylate and inhibit TOR activity, leading to the suppression of growth. Moreover, PYLs, the receptors of ABA, are involved in this regulatory mechanism. In conditions of high nutrient availability, TOR represses ABA signaling and responds to stress stimuli by phosphorylating PYLs, thereby promoting plant growth (Fig. 4) [83].

TOR can also negatively regulate plant immunity [104]. In tomato, the inhibition of TOR has been shown to stimulate the activation of immune responses and confer decreased susceptibility to multiple pathogens through a salicylic acid (SA)-dependent mechanism [104]. Maf1 is a highly conserved RNA polymerase III (Pol III) repressor, playing an important role in regulating protein translation [105]. In citrus plants, Maf1 has been identified as a canker elicitor-binding protein and it inhibits cell growth associated with canker development. Research has found that TOR can regulate Maf1 in plants, and treatment with TOR inhibitors induces dephosphorylation of Maf1, thereby suppressing canker development [105] (Fig. 4). TOR has been identified as the main negative regulator of autophagy [106]. Autophagy has been shown to promote plant resistance to various stressors, such as nutrient deficiency, oxidative stress, and salt and drought stress. These stresses activate autophagy via TOR, and TOR overexpression under these stresses has been found to significantly reduce stress-induced autophagy [107]. In plants, ATG1/13 (autophagy-related 1/13 kinase complex) were the substrates of TOR, which were phosphorylated by TOR protein kinase. Conversely, the dephosphorylation of ATG1/13 enhances autophagy in plants [108, 109] (Fig. 4).

Temperature impacts the activity of TOR too. Under cold stress, TOR kinase activity undergoes transient inhibition, indicating that TOR inhibition represents a significant network for plants to resist cold stress [110]. Moreover, hypersensitivity to cold conditions has been observed in TOR-RNAi lines [111]. Furthermore, high temperatures also exert an influence on TOR. Decreased seedling survival has been associated with both the downregulation of TOR and treatment with the TOR inhibitor AZD8055. Researchers found that overexpression of TOR improved the expression of heat shock genes and seedling survival rates following recovery from heat stress treatments [112]. Additionally, TOR has a positive regulatory role in response to drought and osmotic stresses. When the *Arabidopsis* TOR gene was expressed in rice, it led to improved water-use efficiency, growth, and yield under conditions of limited water availability [113]. These findings collectively show that TOR can mitigate the adverse effects of drought or osmotic stress on the plant [55] (Fig. 4).

## Conclusions and outlook

HXK, SnRK1, and TOR are all pivotal proteins involved in sugar signaling and growth regulation. Ongoing research is being conducted to investigate the roles and functions of these three proteins. Moreover, intricate interconnections have been observed between HXK, SnRK1, and TOR. It is widely acknowledged that TOR and SnRK1 closely interact and maintain a delicate balance in coordinating various metabolic processes (Fig. 4). SnRK1.1 interacted with and phosphorylated RAPTOR1B to inhibit TOR activity [114]. Additionally, in *Arabidopsis* an interaction has been observed between TOR and SnRK1α1, which was found to be enhanced 2-fold upon treatment with ABA [17]. Notably, investigations in grape have revealed that TOR interacts with SnRK1.1 to regulate the expression of genes which are involved in sugar metabolism [115]. SnRK1 forms a complex with SnRK2 and PP2C, resulting in the dissociation of TOR and subsequent activation of early developmental processes. Conversely, during stress conditions, PP2C associates with PYR/PYL, leading to the liberation of SnRK1 and SnRK2, which subsequently activate stress response mechanisms. Additionally, SnRK1.1 has been found to interact with the TOR protein, thereby exerting inhibitory effects on TOR [17, 115]. Simultaneously, under stress conditions, SnRK2 phosphorylates RAPTOR, impeding growth by suppressing TOR activity [83].

SnRK1 and FLZ8 are both critical proteins in negative feedback regulation. The TOR–FLZ8–SnRK1 signaling axis helps plants adapt to environmental changes continuously [116]. A recent study revealed that TOR and SnRK1 are both involved in controlling light-regulated splicing events [117]. Concurring activities of these two energy sensors are indispensable for proper regulation of gene expression and seedling development [117]. TOR and SnRK1 both regulate LR formation. T6P regulates LRs at the center of a regulatory hub linking with energy homeostasis through TOR and SnRK1 [103]. Within the plant system, TOR serves as a pivotal sensing component that integrates cues related to nutrients, energy availability, and environmental factors to finely orchestrate growth and development [1]. In contrast, SnRK1 assumes a pivotal role in perceiving nutrient deprivation and stress signals, leading to the promotion of catabolic processes while concurrently inhibiting anabolic processes and growth [1]. SnRK1 and TOR function synergistically to govern the dynamic equilibrium of plant energy homeostasis [17, 51, 83, 115].

In recent years, some studies have revealed the relationship between HXK and SnRK1. In *Setaria viridis*, the expression patterns of HXK and SnRK1 were found to be distinctly influenced by high and low light conditions [118]. Additionally, it was observed that low light and sugar depletion affected the expression of pivotal target genes regulated by HXK and SnRK1, whereas high light and substantial sugar accumulation resulted in the inhibition of the SnRK1 pathway [118]. ACT DOMAIN REPEAT (ACR) proteins have been reported to serve as a repressor in the glucose signaling pathway [119]. The *acr9-1* mutant was hypersensitive to glucose during seedling growth and anthocyanin accumulation [119]. However, *acr9-1/hxk1-3* and *acr9-1/snrk1* double mutants were no longer sensitive to glucose, which indicated that HXK1 and SnRK1 were necessary for the *acr9-1* mutant to be sensitive to glucose. This result suggests that ACR9 may act upstream of the HXK1-SnRK1 signaling module [119]. In *Nicotiana tabacum*, sucrose synergistically regulates the activities of HXK and SnRK1 to regulate the senescence of leaves [120].

Although we have gained considerable knowledge about the regulatory functions of these three proteins individually and made significant research progress and discoveries in terms of energy and stress signaling regulatory factors, there are still many unanswered questions. The interaction between HXK and TOR is currently not well understood, which suggests potential avenues for future research. Exploring the relationship between HXK and TOR could provide valuable insights into the intricate regulatory mechanisms underlying plant physiology. Numerous unresolved questions surrounding HXK, SnRK1, and TOR warrant further investigation. How do these three proteins collaboratively maintain a delicate balance and regulate sugar metabolism alongside the stress response in plants? Is it possible to identify additional substrates of TOR, SnRK1, or HXK, and elucidate their functions within plant signaling pathways? Beyond their involvement in sugar metabolism and stress response, are there other shared roles that these three proteins play?

TOR, SnRK1, and HXK are three genes that play important roles in plant growth, metabolic regulation, and responses to environmental stress. Therefore, the application and regulation of these genes in horticultural plants hold the potential to improve crop quality, increase yield, and enhance plant resistance to various adversities, providing important theoretical and practical foundations for the improvement and breeding of horticultural plants. Especially, exploring the balanced and coordinated network between TOR and SnRK1 can contribute to future molecular-level regulation of fruit size and quality in horticultural crops, improving the quality characteristics of horticultural plants, such as fruit texture, color, and flavor. Addressing these inquiries concerning TOR, SnRK1, and HXK will undoubtedly contribute to the advancement of plant science. It is plausible that future advancements in manipulating the expression levels of these three proteins may offer a means to regulate horticultural crops.

## Author contributions

Y.Z. wrote and approved the manuscript. GS.L. contributed to the revision of this review.

## Data availability

Not applicable.

## Conflict of interest

The authors declare no conflicts of interest.
